# Analysis of basal serum TSH, FT3, and FT4 levels based on age, sampling time in women with infertility

**DOI:** 10.1186/s12905-021-01453-8

**Published:** 2021-08-28

**Authors:** Yuchao Zhang, Wenbin Wu, Yanli Liu, Xingling Wang, Yichun Guan, Liting Jia

**Affiliations:** 1 Department of Reproductive Medicine, The Third Affiliated Hospital of Zhengzhou University, No. 7 Kangfuqian Street, Erqi, Zhengzhou, Henan China; 2 Neonatal Screening Center, The Third Affiliated Hospital of Zhengzhou University, Zhengzhou, Henan China

**Keywords:** Thyroid stimulating hormones (TSH), Free triiodothyronine (FT3), Free serum thyroxine (FT4), Infertile factors, Female age, Sampling time

## Abstract

**Background:**

To analyze the characteristics of basal thyroid hormone levels in infertile women consulting for assisted reproductive technology (ART) treatment.

**Methods:**

This was a retrospective study. Serum TSH, FT3 and FT4 levels of women seeking ART consultation were tested routinely. Analyses were performed based on age and sampling time. One-way ANOVA or Kruskal-Wallis rank sum test was used to compare the continuous data among the groups, and the chi-square test or Fisher’s exact test was used to compare categorical data where appropriate.

**Results:**

A total of 6426 women were initially included in the study. After exclusion criteria were applied, the remaining 4126 women were categorized into different groups. The prevalence of subclinical hypothyroidism significantly decreased with age and sampling time, from 21.09 to 11.91% and from 28.57 to 10.67%, respectively (*P* < 0.001, respectively). Mean serum TSH, FT3, and FT4 levels decreased significantly with age (*P* = 0.017, < 0.001, < 0.001, respectively). In the context of sampling time, TSH levels from early in the morning were significantly higher (*P* < 0.001), while FT4 and FT3 levels were similar in different groups (*P* = 0.258, 0.300, respectively).

**Conclusions:**

The prevalence of subclinical hypothyroidism significantly decreased with increasing age and sampling time, as did the serum TSH levels. Even though, the establishment of reference interval of TSH level based on age or sampling time was not recommended. Full consideration of age and sampling time should be carefully taken before initiation of treatment.

## Background

Thyroid dysfunction is an important factor that adversely affects the outcome of pregnancy and ART, and affect 2–3% of pregnant women [1]. Thyroid hormones which are the most biomarkers for the diagnosis of thyroid function, are critical for substance metabolism, energy production, early placental formation and neurological development. Treatment of thyroid dysfunction tremendously improves infertility treatment outcomes such as increased implantation rate, decreased miscarriage rate, and increased delivery rate. However, current opinions on whether universal screening for thyroid function should be performed before or during pregnancy are still controversial. We agree with the suggestion that recommendation of universal screening for women undergo infertility treatment should base on the prevalence of thyroid dysfunction in the population with infertility. The American Association of Clinical Endocrinology (AACE) did not recommend general screening for thyroid function in women preparing for pregnancy (or the application of ART) or those who were already pregnant [2]. On the other hand, the guidelines released by the American Thyroid Association (ATA) and the American Society for Reproductive Medicine (ASRM) both recommended screening for TSH in infertile women [3, 4], as did the expert consensus released by the Chinese Medical Association (CMA) [5]. However, the prevalence of thyroid disease in infertile women has not been clearly established; for example, the reported prevalence of subclinical hypothyroidism ranges from 2.3 to 16.7% in infertile women. In addition, it has been well recognized that serum TSH levels are affected by various factors such as age, gender, iodine intake, and most importantly circadian rhythms. When the blood samples are collected on the early morning (before 9:00), TSH levels are significantly higher than that of the later morning from the same patients [6, 7]. Although well-designed studies have claimed that TSH reached the nocturnal peak around 2:00 to 4:00, and met the lowest level between 19:00 and 20:00, patients in our Department of Reproductive Medicine usually have the blood samples collected from 7:00 on the morning to 12:00 at noon, during which period the TSH levels are supposed to decline with time regardless of the pulse release every 2–4 h. As expected, when one euthyroid women is sampled early in the morning, the TSH levels may exceed the upper limit of the reference range, which helps the clinician to make the diagnosis of SCH.

To understand the thyroid status of infertile women in our region, and what factors may impact on the assessment of thyroid function, this study retrospectively analyzed the serum TSH, FT3 and FT4 levels based on age and sampling time of infertile women who received ART counseling in the Department of Reproductive Medicine.

## Methods

This was a retrospective study, which initially included the women who came to the Department of Reproductive Medicine of the Third Affiliated Hospital of Zhengzhou University for ART counseling from September 2018 to December 2019. Those with any of the following criterion were excluded: (1) the results were obtained from reexamination; (2) the data of sampling time, age, or serum hormones levels such as TSH, FT3, and FT4 were missing; (3) the results were obtained a year long before the application of ART; (4) the results did not show the classic patterns of thyroid diseases; (5) the patients visited the department for second or more treatment cycles.

The subjects were divided into five groups based on their ages: <25 years, 26–30 years, 31–35 years, 35–40 years, and > 40 years; and into five groups based on the sampling time: 7:00–8:00, 8:00–9:00, 9:00–10:00, 10:00–11:00, and 11:00–12:00.

All procedures were in accordance with the institutional review board on human experimentation and with the Helsinki Declaration of 1964 and its later amendments. The informed consent was waived by the Institutional Review Board of the Third Affiliated Hospital of Zhengzhou University.

Before venipuncture, the patients were required to stay calm for 30 min at least. All fasting blood samples were collected from patients on the 2nd–4th morning of menstruation. The nurses were well trained for the venipuncture. Specifically, after careful check of the information, the nurse firstly placed a tourniquet 5–10 cm above the elbow, and asked the patient to make a fist. If the vein was obviously raised, the nurse performed strict sterilization with routine aseptic technique, and then fixed the lower end of the vein with the left thumb, and meantime held the needle with her thumb and index finger. Next, the nurse made the needle and the skin at an angle of 30°–50°, and quickly penetrated the skin from the center of the blood vessel, and puncture the needle at an angle of 15°–20°. Finally, the nurse changed the needle body to a parallel angle with the skin, and inserted a little needle along the vein. After collecting enough blood sample, the nurse release the tourniquet and pull out the needle with a sterile cotton coving the puncture point. Samples were centrifuged at 3000 rpm for 10 min after at least half an hour. Then, the serum on the upper layer was used for analysis. The measurements of TSH, FT3 and FT4, as parts of the baseline of endocrine hormones for first visitors, were conducted by electrochemical luminescence (ECLIA) on Cobas 8000 (Roche Diagnostics, Germany). The provided TSH reference range is 0.27–4.2 mIU/L, the FT3 reference range is 3.1–6.8 pmol/L, and the FT4 reference range is 12.0–22.0 pmol/L. Daily internal quality control and yearly external quality control were carried out by request.

The criteria for overt hyperthyroidism (Ohyper) were: TSH < 0.27 mIU/L, FT4 > 22.0 mIU/L, and/or FT3 > 6.8 mIU/L. The criteria for subclinical hyperthyroidism were (Shyper): TSH < 0.27 mIU/L, FT4 levels were in the reference ranges. The criteria for overt hypothyroidism (Ohypo) were: TSH > 4.20 mIU/L and FT4 < 12.0 mIU/L. The criteria for subclinical hypothyroidism (Shypo) were: TSH > 4.20 mIU/L, and FT4 levels were in the reference ranges.

Continuous data were expressed as the mean ± standard deviation. One-way ANOVA or Kruskal-Wallis rank sum test was used for comparison among the groups where appropriate. Categorical data were expressed in number (percentages), and the chi-square test or Fisher’s exact test was used for comparison where appropriate. The statistical software was SPSS 22.0, and *P* < 0.05 was considered as a significant difference among the groups. And adjusted *P* < 0.003 was considered as a significant difference when performing the post hoc test.

## Results

### General characteristics and distribution of thyroid function of the included population

During the study period, 8486 thyroid tests (TSH, FT3, and FT4) were obtained from 6426 infertile women of childbearing age at the time of ART counseling. Finally this study employed 4126 women who met the included criteria, and were divided into subgroups by age and sampling time. The prevalence of SHYPO decreased significantly with age and sampling time (*P* < 0.001, respectively), while the proportion of euthyroid women increased significantly (*P* < 0.001, respectively). (Tables 1, 2)

**Table 1:** Distribution of thyroid function based on age [N (%)]

Age	N	Ohyper^a^	Ohypo^a^	Shyper^a^	Shypo^b^	Normal^b^
≤ 25	455	1 (0.22%)	1 (0.22%)	2 (0.43%)	96 (21.09%)	355 (78.02%)
26–30	1703	5 (0.29%)	13 (0.29%)	13 (0.76%)	325 (19.08%)	1347 (79.09%)
31–35	1224	5 (0.40%)	4 (0.40%)	2 (0.16%)	178 (14.54%)	1035 (84.55%)
36–40	551	3 (0.54%)	3 (0.54%)	3 (0.54%)	87 (15.78%)	455 (82.57%)
> 40	193	1 (0.51%)	2 (0.51%)	1 (0.51%)	23 (11.91%)	166 (86.01%)
*P*		0.747	0.335	0.196	< 0.001	< 0.001

^a^Fisher’s exact test

^b^Chi-square test

**Table 2:** Distribution of thyroid function based on sampling time [N (%)]

Sampling time	N	Ohyper^a^	Ohypo^a^	Shyper^a^	Shypo^b^	Normal^b^
07:00–08:00	434	1 (0.23%)	4 (0.23%)	0 (0%)	124 (28.57%)	305 (70.27%)
08:00–09:00	1081	4 (0.37%)	4 (0.37%)	6 (0.55%)	243 (22.47%)	824 (76.22%)
09:00–10:00	1248	2 (0.16%)	6 (0.16%)	9 (0.72%)	195 (15.62%)	1036 (83.01%)
10:00–11:00	829	4 (0.48%)	7 (0.48%)	4 (0.48%)	90 (10.85%)	724 (87.33%)
11:00–12:00	534	4 (0.74%)	2 (0.74%)	2 (0.37%)	57 (10.67%)	469 (87.82%)
*P*		0.348	0.486	0.500	< 0.001	< 0.001

^a^Fisher’s exact test

^b^Chi-square test

### Serum TSH, FT3 and FT4 levels based on age and sampling time

The serum TSH, FT3 and FT4 levels of 4088 women were analyzed after 38 women with overt thyroid dysfunction (significant abnormalities in serum TSH, FT3, and FT4 levels) were excluded. The results showed that the mean serum TSH, FT3, and FT4 levels decreased significantly with age (Table 3). In the context of sampling time, TSH levels from early in the morning were significantly higher (*P* < 0.001), while FT4 and FT3 levels were similar in different groups (*P* = 0.258, 0.300, respectively) (Table 4)

**Table 3:** Comparison of TSH, FT3 and FT4 levels in different age groups [mean (SD)]

Age	N	TSH (IU/L)^a^	FT4 (pmol/L)^a^	FT3 (pmol/L)^b^
≤ 25	355	2.45 (0.88) ^#^	16.45 (1.95)	5.03 (0.49) ^#^
26–30	1347	2.43 (0.87)	16.37 (1.90)	4.84 (0.52) ^#^
31–35	1035	2.36 (0.86)	16.21 (1.83)	4.71 (0.50) ^#^
36–40	455	2.28 (0.83)	16.07 (1.85)*	4.66 (0.51) ^#^
> 40	166	2.38 (0.89)	15.69 (1.79) ^#^	4.56 (0.43) ^#^
*P*		0.017	< 0.001	< 0.001

^a^ANOVA test

^b^Kruskal–Wallis rank sum test

^#^Indicates a significant differences compared with any other groups

*Indicates significant differences compared with first 3 groups

**Table 4:** Comparison of TSH, FT3 and FT4 levels in different sampling time groups [mean (SD)]

Sampling time	N	TSH (IU/L)^a^	FT4 (pmol/L)^a^	FT3 (pmol/L)^b^
07:00–08:00	355	2.62 (0.88)^#^	16.22 (2.00)	4.80 (0.49)
08:00–09:00	1347	2.50 (0.85) ^#^	16.16 (1.83)	4.76 (0.53)
09:00–10:00	1035	2.40 (0.86) ^#^	16.29 (1.91)	4.78 (0.53)
10:00–11:00	455	2.27 (0.86)*	16.26 (1.88)	4.77 (0.51)
11:00–12:00	166	2.19 (0.84)*	16.4 (1.80)	4.82 (0.49)
*P*		< 0.001	0.258	0.300

^a^ANOVA test

^b^Kruskal–Wallis rank sum test

^#^Indicates a significant differences compared with any other groups

*Indicates significant differences compared with first 3 groups.

## Discussion

In this study, we included a population of infertile women who first visited to our department. The thyroid hormones were detected and analyzed based on age and sampling time. Finally, we observed a decline of serum TSH levels and prevalence of Shypo along with age and sampling time.

Thyroid diseases can interfere with the reproductive outcomes of ART, reduce the likelihood of conception, and adversely affect pregnancy outcomes. Overt thyroid dysfunction can be diagnosed and treated in a timely way due to obvious early symptoms and abnormal serum biomarkers. The FT4 levels of Shypo patients were normal, and treatment was delayed due to the absence of obvious clinical symptoms and signs. Krassas et al. [8] systematically analyzed the relationship between thyroid function and human reproductive health, and claimed that as there were differences in the definitions of Shypo or the included patients, the prevalence of Shypo was obviously different among the studies. However, the prevalence was similar between infertile and ordinary women [9]. Our study showed that the total prevalence of Shypo in 4126 women was 17.18%, which was close to the values reported by Shan et al. (16.7%) [10] and Abalovich et al. (13.9%) [11]. Ehrenkranz et al. [12] addressed the alteration of serum TSH levels corresponding to circadian rhythm, age, and gender, and suggested that “in order to avoid incorrectly identifying TSH values as abnormal, the TSH reference range, needed to take into account the subject’s age and the time of day when the blood sample was drawn”.

Plenty studies provided consistent conclusion that age was an independent factor that influenced serum TSH levels, and suggested to establish age-specific, especially elderly-specific TSH levels in order to correctly access the thyroid state. Both Ehrenkranz et al. [12] and Zhai et al. [13] had established age-specific TSH reference intervals. However, either 10 year-intervals or 20 year-intervals were based on to divide the population into different groups, which might obscure the potential impact of age on serum TSH levels. In this study, we found that the prevalence of SHYPO decreased significantly with increasing age, from 21.09% among women less than 25 years old to 11.91% in women older than 40 years old, which was inconsistent with the reports from other countries [14, 15]. The study conducted by Zhai et al. [13] included the younger population (both men and women) aged from 19 years old to 44 years old, and claimed slightly decreased median TSH levels from 2.50 to 2.32 mIU/L, regardless of significantly increased TSH levels in the old reference population. The discrepancy in terms of TSH levels in the younger population between Zhai et al. [13] and ours may be explained by the gender and age intervals. Moreover, Chen et al. [16] demonstrated similar observation among included healthy female population aged 20–40 years old. Even though, our study reported very slightly decreased TSH levels from young to advanced reproductive age. We believed there were little clinical significance in establishing age-specific TSH reference intervals for younger (in contrast to the elderly) infertile women.

Karmisholt et al. [17] claimed that only when the difference between two TSH results in the same Shypo patient (TSH between 4.2 and 12.0 mU/L) is no less than 40% in the absence of treatment could it truly reflect a change in TSH. Therefore, a single detection of TSH might not reflect thyroid problems in patients. There were other studies reporting that the secretion of TSH had a circadian rhythm, which started to rise in the afternoon and peaking after midnight. Chen et al. [6] demonstrated an obvious circadian rhythm of serum TSH levels in 30 healthy people instead of patients with Alzheimer’s disease who suffer from day-time agitation, night-time insomnia and restlessness. However, patients from the Department of Reproductive Medicine were mostly have the blood samples drawn in the morning, during which period, the TSH levels were supposed to decrease with time. In this study, blood samples were collected from 7:00 in the morning to 12:00 at noon from infertile women attending our department for ART consultation. We found that the prevalence of Shypo decreased significantly with sampling time, from 28.57% among women who have blood drawn early in the morning to 10.67% later in the morning, with TSH levels decreased from 2.62 to 2.19 mIU/L. Ideally, in order to prove circadian changes in TSH serum concentration, we need to test the same patients in different times of the day. This study, regardless of its relative short time period, observed a decline of TSH levels along with time in the morning. Alternative explanations to the finding of different rates of pathological results in different hours of the day in a cohort study could be random, and it could also be the result of patient with Shypo suffering from insomnia and therefore showing up at the clinic at an earlier hour.

According to the prevalence of Shypo reported by Shan et al. [10] approximately 18% patients who have their blood samples drawn in the early morning would be misdiagnosed as Shypo. The observation of this study confirmed the impact of sampling time on the measurement of serum TSH levels, and suggested that consideration of sampling time was strongly required for either resetting of TSH reference range, or interpretation of TSH levels before treatment.

Furthermore, a study pointed out that the reference intervals of serum TSH, FT3 and FT4 were set based on the entire population due to the limitations of the detection methodology, so a TSH result falling within the reference range does not necessarily indicate normal thyroid function. Therefore, for patients with unexplained infertility or high risks of thyroid disease, it is necessary to monitor TSH levels to understand the real status of thyroid function.

There were some limitations in this study. First, this was a retrospective study including a specific population. Second, thyroid globulin antibody and thyroid peroxidase antibody may affect serum TSH levels but were not retrospectively analyzed. Martin et al. [18] reported that the prevalence of Shypo decreased from 2.4% in the thyroid disease-free population aged 12–19 years old to 1.8% in the thyroid disease-free population aged 30–39 years old, and increased ever since 40 years old when thyroid antibodies were negative and cutoff value of TSH was 4.5 mIU/L. However, the alteration trend of prevalence of Shypo did not change when thyroid antibodies were not taken into consideration. Finally, other chronic diseases that may affect thyroid function were missing.

Nevertheless, the purpose of this study was to obtain a full understand of the basic status of thyroid function in infertile women. We found that among them, the prevalence of Shypo decreased significantly with age and sampling time, so as serum TSH levels, and that FT3 levels also decreased significantly with age. Establishment of reference intervals of FT4 and FT3 based on age and sampling time was unnecessary.

## Conclusions

Age and sampling time were non-negligible factors that may impact on TSH levels; full consideration of age and sampling time should be taken before initiation of treatment.

## Data Availability

The original data are available to all readers upon request. Please contact the first author at yuchao1988@yeah.net for such supporting data.

## Abbreviations

ART: assisted reproductive technology
TSH: thyroid-stimulating hormones
FT3: free triiodothyronine
FT4: free serum thyroxine
AACE: American Association of Clinical Endocrinology
ATA: American Thyroid Association
ASRM: the American Society for Reproductive Medicine
CMA: Chinese Medical Association
ECLIA: electrochemical luminescence
Shypo: subclinical hypothyroidism
Shyper: subclinical hyperthyroidism
Ohypo: overt hypothyroidism
Ohyper: overt hyperthyroidis
